# Interspecies gene function prediction using semantic similarity

**DOI:** 10.1186/s12918-016-0361-5

**Published:** 2016-12-23

**Authors:** Guoxian Yu, Wei Luo, Guangyuan Fu, Jun Wang

**Affiliations:** College of Computer and Information Sciences, Southwest University, Chongqing, China

**Keywords:** GO annotations, Semantic similarity, Gene function prediction, Interspecies

## Abstract

**Background:**

Gene Ontology (GO) is a collaborative project that maintains and develops controlled vocabulary (or terms) to describe the molecular function, biological roles and cellular location of gene products in a hierarchical ontology. GO also provides GO annotations that associate genes with GO terms. GO consortium independently and collaboratively annotate terms to gene products, mainly from model organisms (or species) they are interested in. Due to experiment ethics, research interests of biologists and resources limitations, homologous genes from different species currently are annotated with different terms. These differences can be more attributed to incomplete annotations of genes than to functional difference between them.

**Results:**

Semantic similarity between genes is derived from GO hierarchy and annotations of genes. It is positively correlated with the similarity derived from various types of biological data and has been applied to predict gene function. In this paper, we investigate whether it is possible to replenish annotations of incompletely annotated genes by using semantic similarity between genes from two species with homology. For this investigation, we utilize three representative semantic similarity metrics to compute similarity between genes from two species. Next, we determine the *k* nearest neighborhood genes from the two species based on the chosen metric and then use terms annotated to *k* neighbors of a gene to replenish annotations of that gene. We perform experiments on archived (from Jan-2014 to Jan-2016) GO annotations of four species (Human, Mouse, Danio rerio and Arabidopsis thaliana) to assess the contribution of semantic similarity between genes from different species. The experimental results demonstrate that: (1) semantic similarity between genes from homologous species contributes much more on the improved accuracy (by 53.22%) than genes from single species alone, and genes from two species with low homology; (2) GO annotations of genes from homologous species are complementary to each other.

**Conclusions:**

Our study shows that semantic similarity based interspecies gene function annotation from homologous species is more prominent than traditional intraspecies approaches. This work can promote more research on semantic similarity based function prediction across species.

**Electronic supplementary material:**

The online version of this article (doi:10.1186/s12918-016-0361-5) contains supplementary material, which is available to authorized users.

## Background

Gene products, both proteins and RNAs, play crucial functions in many if not all, life processes, such as metabolism, signal transduction and hormonal regulation. Comprehensively annotating their biological functions is a crucial link in the development of drugs, vaccines, bio-chemicals and disease analysis [1–5]. However, rapidly accumulated genomic and proteomic data result in a continually expanding function-annotation gap for newly discovered genes and their products, since it is time consuming, expensive and low throughput to annotate them by wet-lab techniques. Furthermore, the experimental ethics involving human and animals, research interests of biologists and experimental techniques also bias the functional annotations of genes [6–9]. Therefore, automatically and efficiently annotating the functions of genes via computational techniques becomes one of the fundamental tasks in the post-genome era. To combat with this task, some approaches utilize amino acids and structure of proteins [10], some methods resort to protein-protein interactions [11], some other techniques take advantage of domains, motifs and pathways [12, 13]. More advanced techniques integrate multiple types of biological data or fuse predictions from multiple classifiers, which are trained on heterogeneous biological data sources [14–18].

Gene Ontology (GO) is a widely used golden standard for functional taxonomy in a species-neutral manner and it aims to unify the representation of gene products functions across different species [8, 19]. GO uses controlled vocabulary to describe terms (each term corresponds to a distinct function) and a direct acyclic graph (DAG) to capture the hierarchical relationship between ontological terms. For identification, each term is accompanied by an alphanumeric symbol (i.e., GO:0008150 (biological process)). If a gene is annotated with a term, then the gene is also annotated with its ancestor terms via any path in GO hierarchy. If a protein is not annotated with a term, the protein should also not be annotated with any of its descendant terms. This rule is recognized as *true path rule* [19, 20].

Gene function prediction can be viewed as a classification task with each function being viewed as a class label. In this way, various classification techniques have been applied to gene function prediction [2]. A protein engages with several different biological activities and carries out different functions. Recent techniques resort to multi-label learning [21] and correlations among functional labels for gene function prediction [13, 17, 18, 22]. Due to resources limitations, experimental protocols and priority of GO consortium, GO annotations of genes are incomplete [6–9, 23]. Given that, some approaches directly target at replenishing missing annotations of incompletely annotated genes [24–26].

Homologous species share a large portion of homologous genes and these genes have similar (or same) functional annotations. Due to research interests and particular types of experiments performed in different model organisms, homologous genes in different species are often annotated with different terms, and annotations of these genes are found to be complementary for each other [9]. Previous approaches often only use the homology information from deoxyribonucleic acid sequences, structure of proteins, pattern of interactions between genes/proteins, and domain composition to transfer annotations of annotated genes to un-annotated ones [27]. For example, Mitrofanova et al. [28] propose a Markov random field based approach to predict gene function that connects protein-protein interactions (PPI) networks of two (or more) different species by using inter-species sequence-homology information. This approach can only apply to a fixed number (≤32) of structured GO terms, and it only takes proteins annotated with these terms into account and exclude a lot of proteins not annotated with any of these terms. To overcome this issue, Benso et al. [29] firstly defined an integrated similarity between proteins using motifs and amino acids of proteins, and then used this similarity to filter out false positive interactions in PPI network. Next, they enriched the filtered PPI network by adding interactions with annotated proteins through sequence alignment, and assigned the most probable terms to a protein by the terms annotated to its interacting partners. However, it is difficult to specify a suitable threshold to filter out false positive interactions and to add uncovered interactions.

Some advanced techniques exploit the hierarchical structure of GO and characteristics of GO annotations for gene function prediction. For example, Valentini [20], Barutcuoglu et al. [22] and Cesa-Bianchi et al. [17] firstly trained a classifier for each term in the hierarchy, and then made use of ontology structure to adjust the predictions for these terms. Lord et al. [30] directly employed the patterns of GO annotations of genes to predict gene function.

Some recent approaches directly exploit semantic similarity between genes from *single* species to predict gene function. Semantic similarity is computed based GO annotations of genes. It is found to be positively correlated with the similarity derived from various biological data and used to predict interactions between proteins [26, 31–33]. Tao et al. [24] and Yu et al. [26] firstly selected neighborhood genes of a gene using a predefined semantic similarity, and then used the annotations of these neighbors to predict missing annotations of the gene. Given these successful applications and complementary GO annotations of genes from homologous species, however, little work has been done to investigate semantic similarity for inter-species gene function prediction.

In this paper, we investigate whether it is possible to perform inter-species gene function prediction by directly using the semantic similarity between genes from two different species, which have homology to some extent. For this purpose, we utilize several representative semantic similarity metrics (i.e., term overlap (TO) [34], best match average (BMA) [35] and simGIC [36]) to measure the semantic similarity between genes, and make use of these metrics for semantic similarity based gene function prediction. We study these metrics’ contributions on improving the accuracy of gene function prediction for two homologous species (*Human* and *Mouse*). In addition, we also include another two species, *Danio rerio* and *Arabidopsis thaliana*, which have lower homology with Human and Mouse. Our investigation discloses that, interspecies gene function prediction using semantic similarity between genes from homologous species (Human and Mouse) outperforms the counterpart based on the semantic similarity between genes from single species alone, and it also performs better than using the semantic similarity between genes from two species with low homology.

## Methods

Our work is motivated by the observation that GO annotations of genes are incomplete [7, 9] and genes from homologous species should have a large portion of similar GO annotations [8, 23]. However, because of experimental ethics and protocols, and research interests of biologists, homologous genes from different species currently are only annotated with some similar GO terms, these genes are also annotated with different terms. These different annotations provide complementary functional clue for genes from another high homology species. For example, as shown in Table S1 of Additional file 1, Human hMAP4K2 (Mitogen activated protein kinase kinase kinase kinases (MAP4K) are protein kinases that participate in the MAP kinase signal transduction cascade) shares 94% sequence identity with its ortholog Map4k2 in Mouse [9]. The overlapped GO annotations of these two proteins account for 81.19% of all the available annotations of these proteins by Jan-2014. hMAP4K3, a paralog of hMAP4K2, has 76.24% overlapped annotations with hMAP4K2. As more experimental evidences available, some terms only annotated to hMAP4K2 are also annotated to Map4k2, and vice versa. By Jan-2016, as more evidences accumulated in the past two years, the overlapped annotations between hMAP4K2 and MAP4K increases to 98.11%, and that between hMAP4K2 and hMAP4K3 rises to 86.79%. GO annotations of these three proteins by Jan-2014 and Jan-2016 are listed in Table S1 of Additional file 1. In addition, the evidence sources of new overlapped annotations from Jan-2014 to Jan-2016 are provided in Table S2 of Additional file 1. These observations indicate GO annotations of homologous genes from two species with high homology are complementary for each other.

**Fig. 1:**
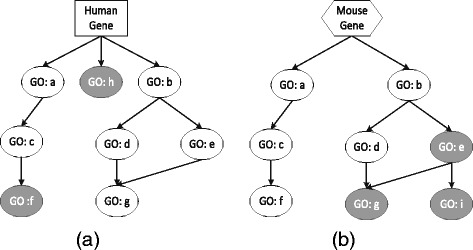
GO annotations of a human gene **a** and a mouse gene **b**. GO terms in white ellipses are the currently available annotations of the gene, and the terms in the gray ellipses are the missing annotations. The human gene should be annotated with ‘GO:f’ and mouse gene missing annotations of ‘GO:e’ and ‘GO:g’. Annotations of these two genes are different but also complementary for each other

Inspired by the aforementioned observation, we want to synthesize the semantic similarity between genes from single species and from two homologous species to predict additional annotations of genes. To better explain our main idea, we provide an illustrative example in Fig. 1. We can see from Fig. 1 that both a Human gene and a Mouse gene are annotated with a set of similar terms. Both of them also lack some annotations, respectively. The Human gene should be additionally annotated with ‘GO:f’ and ‘GO:h’, and the Mouse gene should be additionally annotated with ‘GO: e’, ‘GO:g’ and ‘GO: i’. By using the semantic similarity between these two genes from Human and Mouse, we can transfer available GO annotations of the Human gene to the Mouse gene, and thus to replenish the missing annotations of the latter one. Vice versa, we can transfer annotations of the Mouse gene to the Human gene. In this way, we can replenish missing annotations of respective genes by utilizing semantic similarity and complementary GO annotations of genes across species.

### Semantic similarity

Semantic similarity has been widely studied, and various methods have been proposed for quantifying the semantic similarity between genes [32, 33]. These methods fall into rough categories of term-based and entity-based. In general, term-based approaches depend on comparing and combining pairwise terms annotated to two respective genes, while entity-based approaches rely on comparing two sets of terms, each set is associated with a gene [32]. As a node in GO DAG, each term not only includes specific properties, but also connects with other terms by edges with different relationships (is a, part of and regulates). Therefore, there are two types of measures to calculate the similarity between terms in GO, node-based and edge-based. Node-based measures rely on the information of terms themselves, their ancestors, or their descendants. These measures mainly utilize the information content, which estimates a term’s specificity by its frequency annotated to genes [37–39]. Edge-based measures are mainly based on counting the number of edges in the GO DAG between two terms. For example, Pekar et al. [40] computed the proximity between two terms by the length of path from their lowest common ancestor to the root term. Wang et al. [41] suggested a hybrid measure that determines the proximity of two terms based on the locations of these terms in GO hierarchy and the relationships with their ancestor terms.

Entity based semantic similarity metrics calculate similarity between genes by comparing two sets of terms annotated to two respective genes as a whole. Pesquita et al. [32] suggested to divide these metrics into two categories: pairwise and groupwise. Pairwise metrics integrate the proximity between pairwise terms using average [31], maximum combination and best match average (*BMA*) combination [42]. Groupwise metrics directly apply set, graph, or vector based measures to compute the similarity between two collections of terms. For example, Mistry and Pavlidis [34] introduced a set based metric called term overlap (*TO*), which takes the ratio between the number of shared annotations and minimum number of annotations of two genes. In graph-based metrics, terms annotated to a gene can be represented by a subgraph of GO DAG, and graph comparing techniques are used to measure the similarity between genes. For example, *simUI* takes the ratio between the number of common nodes of two subgraphs and the number of union nodes of these two subgraphs [42]. *simGIC* is similar to simUI, but it weights each term by information content of the term [42]. Vector-based metrics represent the associations between a gene and all terms as a vector, with each term corresponding to an entry, and then calculate similarity between genes using vector-based measures, i.e., cosine similarity.

Three representative semantic similarity metrics (TO [34], BMA and simGIC [42]) are adopted to investigate interspecies gene function prediction based on semantic similarity. BMA requires to specify the proximity between pairwise terms and simGIC needs to pre-compute the information content of a term. We choose Lin’s similarity [43] to measure the proximity of pairwise terms for its wide application and fixed scale (between 0 and 1). Lin’s similarity evaluates the proximity of two terms by the information of themselves and of their most specific common ancestor in GO hierarchy. Its formal definition is:

1 $$ tsim(t_{1},t_{2})=\frac{2\times{IC(t_{A})}}{IC(t_{1})+IC(t_{2})}  $$

where *t*_*A*_ is the most informative common ancestor of term *t*_1_ and *t*_2_. *IC*(*t*) is the information content of *t* and can be calculated as:

2 $$ IC(t)=-\log_{2}p(t)  $$

where *p*(*t*) is the frequency of *t* annotated to a gene. Tao et al. [24] and Teng et al. [44] suggested a ontology structure based manner to define the information content of *t* by the number of its descendants in GO hierarchy, instead of its frequency. The structure based information content *IC*_*s*_(*t*) is:

3 $$ {IC}_{s}(t)=\frac{\log_{2}((1+|desc(t)|)/T)}{\log_{2}(1/T)}  $$

where |*desct*(*t*)| is the set of descendant GO terms of *t* and *T* is the total number of terms.

Given two genes *i* and *j*, their GO annotation sets are $\mathcal {T}_{i}$ and $\mathcal {T}_{j}$, respectively. BMA is given by the average proximity between each term in $\mathcal {T}_{i}$ and its most similar term in $\mathcal {T}_{j}$. BMA provides a good balance between the maximum and average approaches, since the latter two approaches are inherently influenced by the number of terms being combined [32]. The formal definition of BMA is:

4 $$ \begin{aligned} {sim}_{BMA}(i,j)=\frac{1}{2} ({AVG}_{t_{1}}({MAX}_{t_{2}}tsim(t_{1},t_{2})+ \\ {AVG}_{t_{2}}({MAX}_{t_{1}}tsim(t_{1},t_{2})) \end{aligned}  $$

where $t_{1}\in {\mathcal {T}_{i}}, t_{2}\in {\mathcal {T}_{j}}$ and *tsim*(*t*_1_,*t*_2_) is the semantic similarity between *t*_1_ and *t*_2_. In this paper, we compute *tsim*(*t*_1_,*t*_2_) using Lin’s similarity with *IC*(*t*) defined by Eq. (2).

TO is a set-based metric [34], it is given by number of terms shared by $\mathcal {T}_{i}$ and $\mathcal {T}_{j}$ divided by the minimum cardinal of $\mathcal {T}_{i}$ and $\mathcal {T}_{j}$. Its formal definition is:

5 $$ {sim}_{TO}(i,j)=\frac{|\mathcal{T}_{i}\cap {\mathcal{T}_{i}|}}{min\left(|\mathcal{T}_{i}|,|\mathcal{T}_{j}|\right)}  $$

simGIC is a graph-based semantic similarity metric [25], it is given by the sum of *IC*(*t*) of each *t* in the intersection of $\mathcal {T}_{i}$ and $\mathcal {T}_{j}$, divided by the number of terms in the union of $\mathcal {T}_{i}$ and $\mathcal {T}_{j}$. Its formal definition is:

6 $$ {sim}_{GIC}(i,j)=\frac{\sum_{t\in \mathcal{T}_{i}\cap \mathcal{T}_{j}} IC(t)}{\sum_{t\in \mathcal{T}_{i}\cup \mathcal{T}_{j}} IC(t) }  $$

where *IC*(*t*) can be calculated by Eq. (3) or Eq. (4), To be different, we call simGIC based on *IC*_*s*_(*t*) in Eq. (4) as *simGICs*.

### Gene function prediction using semantic similarity

Suppose there are two species, $A^{s}=[{A_{1}^{s}}, {A_{2}^{s}},\cdots,A_{N_{s}}^{s}]\in \mathbb {R}^{{N_{s}}\times {T}}$(*s*=1,2) be the gene-term association matrices for genes from these two species, respectively. These *N*_*s*_ genes are annotated with *T* different terms. ${A_{i}^{s}}\in \mathbb {R}^{T}$ represents the associations between the *i*-th gene and *T* terms. ${A_{i}^{s}}(t)=1$ means the gene is annotated with term *t*, and ${A_{i}^{s}}(t)=0$ indicates that it is unknown whether the gene should be annotated with *t* or not.

The semantic similarity between genes is found to be positively correlated with the similarity derived from various types of genomic/proteomic data [31–33, 44, 45]. For example, amino acids sequences, gene expression profiles, protein-protein interactions. Tao et al. [24] and Yu et al. [26] computed the semantic similarity between pairwise genes from the same species and determined the *k* nearest neighborhood genes based on the semantic similarity, and then replenished novel annotations of a gene based on the terms annotated to its neighbors. The probability of term $t \notin \mathcal {T}_{i}$ annotated to the *i*-th gene is voted as follow:

7 $$ p(i,t)=\frac{1}{k}\sum\limits_{j\in{\mathcal{N}_{k}(i)}}A_{j}(t)  $$

where $\mathcal {N}_{k}(i)$ consists of *k* nearest neighbors of the *i*-th gene from the same species. From Eq. (7), we can replenish the missing annotation of the *i*-th gene if its neighbors, who are annotated with *t*. Because of resource limitations, priority of GO curators, experimental ethics and protocols, the GO annotations of its neighborhood genes from the same species may be shallow, incomplete and biased [6, 8, 9]. Equation (7) only accounts for GO annotations of genes from the same species, therefore it can only replenish some missing annotations. On the other hand, GO annotations of genes from homologous species may be annotated with more comprehensive and specific terms.

It is recognized that homologous genes from different species conserve a large portion of similar annotations [8, 9, 46]. Motivated by this fact, we resort to semantic similarity between genes from two species and to predict missing annotations of genes by transferring annotations of genes from two species, instead from single species they belonging to. In this way, if a gene has a small semantic similarity with genes from its own species, it still can have high semantic similarity with genes from another species, and these genes may be just annotated with the missing terms of that gene. Given that, we integrate GO annotations of two species and predict missing annotations of the *i*-th gene as below:

8 $$ p(i,t)=\frac{1}{k_{1}+k_{2}} \sum\limits_{j\in \mathcal{N}_{k_{s}}^{s}(i)} {A_{j}^{s}}(j,t)  $$

where $\mathcal {N}_{k_{1}}^{1}(i)$ denotes the *k*_1_ nearest neighborhood genes of the *i*-th gene from its own species, $N_{k_{2}}^{2}(i)$ denotes the *k*_2_ nearest neighborhood genes from another species. *k*_1_>0, *k*_2_>0 and *k*_1_+*k*_2_=*k*, this setting ensures neighborhood genes from two species instead from single species, and is consistent with Eq. (7). Our following experimental study shows that synergy the semantic similarity between genes from two homologous species can more accurately predict gene function than that from single species alone.

## Results and discussion

### Datasets and experimental setup

To comparatively study the contribution of integrating semantic similarity between genes and GO annotations of genes from two species, we conduct experiment on annotations of genes from Human and Mouse. We downloaded recent GO file [47] (access date: 2016-01-04) that contains hierarchical relationships between GO terms. These terms are organized in three sub-ontology, namely biological process (BP), cellular component (CC) and molecular functions (MF), the terms in each ontology form a DAG. We downloaded historical GO annotation (GOA) file [48] (archived date: 2014-01-20) for each species. GOA file specifies which GO terms are annotated to a given gene products, it follows a convention to annotate a gene with appropriate and as well as specific terms. These annotations are called direct annotations. We applied true path rule to annotate all the ancestor terms of direct annotations of a gene to the same gene. We then made use of these annotations to predict GO annotations of genes. Next, we updated the annotations of these genes using recent GOA files (archived date: 2016-01-04) and utilized updated annotations to assess the quality of prediction. To avoid circular prediction, annotations with evidence code ‘IEA’ (Inferred from Electronic Annotation), ‘NR’ (Not Recorded), ‘ND’ (No biological Data available), or ‘IC’ (Inferred by Curator) were excluded. Myers et al. [49] suggested that terms annotated to too few genes are hard to be validated by wet-lab experiments and of no interests to biologists. Follow this suggestion, we excluded terms annotated to no more than 3 genes in each species.

To investigate whether GO annotations from any species contribute the same for interspecies gene function prediction, we also downloaded GOA files of another two species (*Danio rerio* and *Arabidopsis thaliana*) (archived date: 2014-01-20), and processed available GO annotations of these species in the same way as Human and Mouse. The processed annotations of these four species are revealed in Table 1. From the table, we can find that a number of new annotations have been appended to genes from each species since 2014, and each gene on average is annotated with at least 4 terms.

**Table 1:** Statistics of GO annotations of genes from four species

Species	*♯*genes	CC			MF			BP		
		history	recent	*♯*terms	history	recent	*♯*terms	history	recent	*♯*terms
Human	19158	231057	298776	1160	115066	153722	1999	745989	920663	8696
Mouse	21357	164539	291488	1193	95511	158471	2033	622591	933356	9304
Danio rerio	18776	27434	77539	627	24652	59710	1174	172557	301163	4382
Arabidopsis thaliana	24532	114777	150144	528	42720	64737	1189	197277	281562	3305

*♯*genes is the number of genes in the recent GOA file (archived date: 2016-01-04), *♯*terms is the number of involved terms. ‘history’ is the number of GO annotations of genes from historical GOA file (archived date: 2014-01-20), ‘recent’ is the number of GO annotations of genes from recent GOA files

To assess whether the semantic similarity defined by annotations of genes from two homologous species can improve the accuracy of gene function prediction than that from single species alone, we firstly compute the semantic similarity between genes from single species by a specific metric (i.e., TO, simGIC, BMA), and then employ Eq. (7) to predict functions of genes from the same species. Similarly, we also compute the semantic similarity between genes from two species using the same metric and then employ Eq. (8) to predict functions of genes from two species. To balance the contribution of genes from the same species and from another species, we set *k*_1_=250, *k*_2_=250 and *k*=500 for all the following experiments.

### Evaluation metrics

Various evaluation metrics are used to assess the quality of gene function prediction [2, 25]. Since a gene is often annotated with more than one terms, we adopt three representative multi-label learning evaluation metrics [21]: *MacroAvgF1*, *MicroAvgF1*, *RankLoss*, and two additional metrics *Fmax* [2] and *RAccuracy* [50]. The formal definitions of these widely used metrics are detailed in Additional file 1.

To maintain consistency with other evaluation metrics, we report *1-RankLoss*. Thus, similar to other metrics, the larger the value of *1-RankLoss*, the better the performance is. We would like to remark that these metrics evaluate the quality of function prediction from different aspects. It is difficult for a method always performing better than another one across all these metrics.

### Prediction on archived GO annotations

In this section, we conduct experiments to comparatively and quantitatively study the contribution of semantic similarity between genes from single species, from two species with high (or low) homology. Particularly, we perform intraspecies gene function prediction by computing semantic similarity between genes from Human at first. Then, we utilize annotations of *k* nearest neighborhood Human genes of a Human gene to replenish missing annotations of the gene as Eq. (7). Next, we use updated annotations in the recent GOA file of Human to validate the predictions. We label the intraspecies approach as H →H. For brevity, hereinafter, *H* is short for Human species, *M* is short for Mouse, *D* is short for Danio rerio and *A* is short for Arabidopsis thaliana. Similarly, we perform interspecies gene function prediction by using the same semantic similarity metric between genes from Human and another species. Then, we use the annotations of *k*1 nearest neighborhood genes from Human and *k*_2_ nearest neighborhood genes from another species to predict missing annotations of a Human gene, and validate the predictions by annotations in recent GOA file of Human. We tag these interspecies approaches as M+H →H, D+H →H and A+H →H, respectively. In addition, we also direct use the GO annotations of *k* nearest neighborhood Mouse (Danio rerio or Arabidopsis thaliana) genes of a Human to predict the missing annotations of the Human gene. We tag this kind of approaches as M →H (D →H or A →H). Following the same protocols, we conduct similar experiments on Mouse for intraspecies and interspecies gene function prediction. The recorded experimental results under different semantic similarity metrics are reported in Table 2 (BMA) and Table 3 (TO). The results with other semantic similarities (simGIC and simGICs) are included in Table S3 and Table S4 of Additional file 1.

**Table 2:** Prediction on archived GOA files using *BMA* (see Eq. (4))

		MicroAvgF1	MacroAvgF1	1-RankLoss	Fmax	RAccuracy
CC	H →H	0.8328	0.7203	0.8780	0.8747	0.1542
	M →H	0.8368	0.7125	0.8751	0.8750	0.1602
	M+H →H	**0.8586**	**0.7639**	**0.9808**	**0.8787**	**0.2844**
	D →H	0.8316	0.7197	0.8729	0.8738	0.1536
	D+H →H	0.8524	0.7236	0.9068	0.8625	0.2530
	A →H	0.8259	0.7068	0.8632	0.8532	0.1369
	A+H →H	0.8363	0.7222	0.8797	0.8773	0.1717
	M →M	0.7712	0.6084	0.8588	0.8571	0.1936
	H →M	0.7676	0.6192	0.8580	0.8339	0.1864
	H+M →M	**0.8161**	**0.6590**	**0.9548**	**0.8637**	**0.3518**
	D →M	0.7718	0.6105	0.8416	0.8082	0.1868
	D+M →M	0.8003	0.6160	0.8926	0.8315	0.2963
	A →M	0.7660	0.6341	0.8444	0.8404	0.1761
	A+M →M	0.7713	0.6143	0.8606	0.8523	0.1942
MF	H →H	0.8523	0.8179	0.9192	0.8915	0.1416
	M →H	0.8513	0.8170	0.9145	0.8905	0.1311
	M+H →H	**0.8692**	**0.8372**	**0.9720**	**0.9029**	**0.2399**
	D →H	0.8502	0.8174	0.9123	0.8909	0.1295
	D+H →H	0.8668	0.8355	0.9523	0.8742	0.2259
	A →H	0.8416	0.8207	0.8964	0.8968	0.0793
	A+H →H	0.8490	0.8151	0.9116	0.8894	0.1227
	M →M	0.7654	0.6849	0.8755	0.8656	0.1344
	H →M	0.7601	0.6821	0.8797	0.8545	0.1396
	H+M →M	0.7784	**0.7081**	**0.9248**	**0.8779**	0.1801
	D →M	0.7607	0.6891	0.8592	0.8369	0.1287
	D+M →M	**0.7841**	0.7072	0.9200	0.8580	**0.2013**
	A →M	0.7534	0.6880	0.8553	0.8607	0.0876
	A+M →M	0.7639	0.6716	0.8712	0.8551	0.1264
BP	H →H	0.8373	0.7979	0.9507	0.8012	0.2044
	M →H	0.8346	0.7943	0.9489	0.7981	0.1912
	M+H →H	**0.8450**	**0.8055**	**0.9690**	**0.8381**	**0.2421**
	D →H	0.8368	0.8027	0.9568	0.8031	0.2020
	D+H →H	0.8368	0.7978	0.9496	0.8093	0.2018
	A →H	0.8290	0.7903	0.9239	0.7799	0.1641
	A+H →H	0.8325	0.7839	0.9308	0.7944	0.1809
	M →M	0.7812	0.6965	0.9350	0.7905	0.1855
	H →M	0.7842	0.6987	0.9401	0.7863	0.1965
	H+M →M	**0.7947**	**0.7134**	**0.9609**	**0.8270**	**0.2357**
	D →M	0.7816	0.7036	0.9348	0.7885	0.1867
	D+M →M	0.7830	0.7108	0.9423	0.7875	0.1923
	A →M	0.7768	0.6929	0.9027	0.7594	0.1692
	A+M →M	0.7779	0.6871	0.9183	0.7807	0.1733

H →H directly uses GO annotations of Human to predict annotations of Human genes. M →H only employs annotations of genes from Mouse to predict annotations of Human genes. M+H →H uses GO annotations of genes from Mouse and Human to predict annotations of Human genes. D+H →H uses annotations of genes from Danio rerio and Human to predict annotations of Human genes. A+H →H uses annotations of genes from Arabidopsis thaliana and Human to predict annotations of Human genes. M →M, H+M →M, D+M →M and A+M →M follow the similar protocol, but predict annotations of Mouse genes. The data in boldface is the statistically significant best among these comparing methods for a particular target species, and the significance is checked by paired t-test at 95% confidence intervals

**Table 3:** Prediction on archived GOA files using *TO* (see Eq. (5))

		MicroAvgF1	MacroAvgF1	1-RankLoss	Fmax	RAccuracy
CC	H →H	0.8374	0.7212	0.8968	0.8729	0.1773
	M →H	0.8351	0.7241	0.8969	0.8743	0.1762
	M+H →H	**0.8586**	**0.7641**	**0.9771**	**0.8751**	**0.2845**
	D →H	0.8351	0.7322	0.8941	0.8693	0.1662
	D+H →H	0.8512	0.7476	0.9422	0.8654	0.2469
	A →H	0.8317	0.6982	0.8832	0.8860	0.1488
	A+H →H	0.8366	0.7223	0.8962	0.8726	0.1732
	M →M	0.7765	0.6075	0.8826	0.8526	0.2122
	H →M	0.7805	0.6130	0.8836	0.8295	0.2166
	H+M →M	**0.8132**	**0.6547**	**0.9597**	**0.8665**	**0.3418**
	D →M	0.7726	0.6142	0.8659	0.8320	0.2092
	D+M →M	0.7993	0.6357	0.9252	0.8384	0.2928
	A →M	0.7758	0.6278	0.8700	0.8324	0.2105
	A+M →M	0.7770	0.6088	0.8807	0.8447	0.2142
MF	H →H	0.8569	0.8228	0.9293	0.8952	0.1687
	M →H	0.8542	0.8213	0.9262	0.8941	0.1527
	M+H →H	**0.8711**	**0.8382**	**0.9763**	**0.9064**	**0.2510**
	D →H	0.8524	0.8348	0.9413	0.8717	0.1426
	D+H →H	0.8606	0.8349	0.9588	0.8979	0.1901
	A →H	0.8456	0.8225	0.9124	0.8941	0.1026
	A+H →H	0.8535	0.8181	0.9260	0.8933	0.1489
	M →M	0.7756	0.6946	0.8985	0.8692	0.1697
	H →M	0.7804	0.6957	0.9096	0.8569	0.1677
	H+M →M	**0.7851**	**0.7104**	**0.9374**	**0.8806**	**0.2051**
	D →M	0.7695	0.6811	0.8963	0.8602	0.1538
	D+M →M	**0.7851**	0.7082	0.9356	0.8731	**0.2051**
	A →M	0.7635	0.6941	0.8816	0.8588	0.1249
	A+M →M	0.7752	0.6840	0.8993	0.8616	0.1683
BP	H →H	0.8460	0.8019	0.9605	0.8729	0.2472
	M →H	0.8428	0.7998	0.9586	0.7818	0.2316
	M+H →H	**0.8500**	**0.8071**	**0.9745**	**0.8751**	**0.2664**
	D →H	0.8385	0.8036	0.9605	0.7901	0.2101
	D+H →H	0.8443	0.8016	0.9613	0.7877	0.2387
	A →H	0.8314	0.7943	0.9333	0.7591	0.1755
	A+H →H	0.8389	0.7933	0.9520	0.7813	0.2120
	M →M	0.7960	0.7101	0.9519	0.7813	0.2405
	H →M	0.7980	0.7073	0.9532	0.7767	0.2481
	H+M →M	**0.8024**	**0.7163**	**0.9677**	**0.8244**	**0.2643**
	D →M	0.7886	0.7137	0.9508	0.7756	0.2129
	D+M →M	0.7832	0.7059	0.9410	0.7765	0.2318
	A →M	0.7795	0.7020	0.9158	0.7373	0.1791
	A+M →M	0.7723	0.6923	0.9326	0.7716	0.2106

H →H directly uses GO annotations of Human to predict annotations of Human genes. M →H only employs annotations of genes from Mouse to predict annotations of Human genes. M+H →H uses GO annotations of genes from Mouse and Human to predict annotations of Human genes. D+H →H uses annotations of genes from Danio rerio and Human to predict annotations of Human genes. A+H →H uses annotations of genes from Arabidopsis thaliana and Human to predict annotations of Human genes. M →M, H+M →M, D+M →M and A+M →M follow the similar protocol, but predict annotations of Mouse genes. The data in boldface is the statistically significant best among these comparing methods for a particular target species, and the significance is checked by paired t-test at 95% confidence intervals

From these tables, we can observe that M+H →H always gets better results than H →H and M →H, irrespective of the semantic similarity metrics (TO, BMA, simGIC and simGICs). Taking evaluation metric *RAccuracy* in Table 2 for example, M+H →H on average improves H →H by 53.22% and M →H by 62.38%. M+H →H utilizes GO annotations of Human and Mouse to compute the semantic similarity between genes by a chosen metric, and then uses the annotations of *k* nearest neighborhood genes (including *k*_1_ Human genes and *k*_2_ Mouse genes) of a Human gene to predict annotations of the gene. In contrast, H →H only employs semantic similarity between genes from Human species, and the annotations of *k* nearest neighborhood Human genes of a gene to predict GO annotations of the target Human gene. M →H only utilizes the annotations of *k* nearest neighborhood Mouse genes of a Human gene to predict GO annotations of the target Human gene. D+H →H always outperforms D →H and A+H →H outperforms A →H. From this observation, we can say GO annotations of genes from two different species should work together for interspecies gene function prediction.

D+H →H and A+H →H follow the same procedures as M+H →H to predict GO annotations of genes from Human, except they synergy GO annotations of Danio rerio (or Arabidopsis thaliana) with those of Human. These two approaches to M+H →H. D →H and A →H follow the same protocols as M →H to predict GO annotations of genes from Human, and they are outperformed by M →H and sometimes by H →H. In actual fact, Tao et al. [24] and Yu et al. [26] also adopt similar techniques as H →H (or M →M) for intraspecies gene function prediction. From these results, we can say that interspecies gene function prediction based on semantic similarity from two species with high homology is more prominent than traditional intraspecies approaches. Compared with Mouse, Danio rerio has lower homology (about 85%) with Human, and Arabidopsis thaliana has even lower homology with Human. Given that, M+H →H performs better than D+H →H, and it performs even more better than A+H →H. D+H →H also produces better results than A+H →H. These results show that synergy GO annotations of two species with high homology contributes much more for interspecies gene function prediction than synergy GO annotations of two species with low homology.

From these tables, we can find A+H →H often produces similar (or lower) results as H →H. The cause is that Arabidopsis thaliana has the lowest homology with Human among these species. The results on Mouse give the similar observations and lead to the same conclusions. From these comparisons, we can conclude that GO annotations of two species with high homology are more complementary for each other than two species without such high homology.

The largest improvement on *RAccuracy* is CC sub-ontology, followed with MF sub-ontology and then BP sub-ontology. The reason is that the number of involved GO terms and annotations in CC, MF and BP increases one by one, so the complementary effect of GO annotations across species in CC is more prominent than that in MF and BP. Another interesting observation is that, irrespective of TO, BMA, simGIC and simGICs, M+H →H obtains relatively close values for each evaluation metric under every fixed setting. This observation strengthens that our conclusions are independent of the adopted semantic similarity.

To check the difference between M+H →H and H →H, D+H →H and A+H →H based on the results in Tables 2–3 and Tables S3-S4, we use Wilcoxon signed rank test [51, 52] and find that M+H →H significantly performs better than them with *p* value smaller than 10^−10^. We perform the same test to check the difference between H+M →M, M →M, D+H →M and A+H →M. We also find H+M →M works significantly better than them with *p*<10^−9^.

To investigate the effect of GO annotations across sub-ontology, we further combine GO annotations in CC, MF and BP together for function annotation prediction using genes from single species (Human or Mouse) or from two species, and then follow the same protocol as in previous experiments to evaluate the predictions on Human (or Mouse) genes for each sub-ontology. The recorded results using semantic similarity BMA are reported in Table 4. The results using the semantic similarity TO are included in Table S4 of the Additional file 1.

**Table 4:** Prediction on archived GOA files using *BMA* (see Eq. (6)) by combining the GO annotations in CC, MF and BP together and then evaluating in each sub-ontology

		MicroAvgF1	MacroAvgF1	1-RankLoss	Fmax	RAccuracy
CC	H →H	0.8700	0.4416	0.9682	0.8619	0.2057
	M →H	0.8550	0.4407	0.9310	0.8551	0.1963
	M+H →H	**0.8765**	**0.4451**	**0.9791**	**0.9006**	**0.2457**
	D →H	0.8543	0.4372	0.9387	0.8610	0.1626
	D+H →H	0.8666	0.4412	0.9652	0.8773	0.1852
	A →H	0.8424	0.4388	0.8862	0.8595	0.1428
	A+H →H	0.8673	0.4358	0.9518	0.8761	0.1895
	M →M	0.8193	0.4430	0.9487	0.8481	0.1556
	H →M	0.8155	0.4416	0.9475	0.8514	0.1582
	H+M →M	**0.8256**	**0.4507**	**0.9692**	**0.8795**	**0.1853**
	D →M	0.8085	0.4433	0.9289	0.8490	0.1446
	D+M →M	0.8170	0.4474	0.9460	0.8560	0.1452
	A →M	0.7963	0.4258	0.9121	0.8160	0.1157
	A+M →M	0.8162	0.4377	0.9241	0.8385	0.1410
MF	H →H	0.8539	0.4287	0.9569	0.8394	0.1983
	M →H	0.8514	0.4282	0.9468	0.8352	0.1721
	M+H →H	**0.8606**	**0.4312**	**0.9721**	**0.8785**	**0.2349**
	D →H	0.8513	0.4232	0.9507	0.8290	0.1358
	D+H →H	0.8532	0.4294	0.9540	0.8451	0.1945
	A →H	0.8435	0.4217	0.9060	0.8049	0.0921
	A+H →H	0.8453	0.4239	0.9394	0.8187	0.1508
	M →M	0.7980	0.4015	0.9426	0.8066	0.1528
	H →M	0.7963	0.3927	0.9246	0.8001	0.1501
	H+M →M	**0.8078**	**0.4090**	**0.9672**	**0.8629**	**0.1937**
	D →M	0.7596	0.3936	0.9096	0.7748	0.1108
	D+M →M	0.7989	0.4053	0.9427	0.8216	0.1563
	A →M	0.7452	0.3883	0.8856	0.7528	0.0815
	A+M →M	0.7949	0.3984	0.9274	0.7829	0.1395
BP	H →H	0.8376	0.7977	0.9522	0.8023	0.2058
	M →H	0.8320	0.7861	0.9267	0.8134	0.1791
	M+H →H	**0.8450**	**0.8055**	**0.9694**	**0.8390**	0.2421
	D →H	0.8374	0.7917	0.9513	0.8041	0.1948
	D+H →H	0.8370	0.7978	0.9502	0.8098	0.2032
	A →H	0.8248	0.7840	0.8998	0.8119	0.1433
	A+H →H	0.8322	0.7830	0.9328	0.7941	0.1796
	M →M	0.7814	0.6968	0.9372	0.7916	0.1864
	H →M	0.7892	0.6884	0.9384	0.7901	0.1897
	H+M →M	**0.7949**	**0.7132**	**0.9611**	**0.8276**	**0.2363**
	D →M	0.7829	0.6999	0.9364	0.7853	0.1818
	D+M →M	0.7820	0.7033	0.9365	0.7910	0.1883
	A →M	0.7694	0.6897	0.9023	0.7769	0.1417
	A+M →M	0.7779	0.6874	0.9199	0.7822	0.1732

H →H directly uses GO annotations of Human to predict annotations of Human genes. M →H only employs annotations of genes from Mouse to predict annotations of Human genes. M+H →H uses GO annotations of genes from Mouse and Human to predict annotations of Human genes. D+H →H uses annotations of genes from Danio rerio and Human to predict annotations of Human genes. A+H →H uses annotations of genes from Arabidopsis thaliana and Human to predict annotations of Human genes. M →M, H+M →M, D+M →M and A+M →M follow the similar protocol, but predict annotations of Mouse genes. The data in boldface is the statistically significant best among these comparing methods for a particular target species, and the significance is checked by paired t-test at 95% confidence intervals

From the these tables, we can find an interesting observation is that H →H in Table 4 (and Table S4) has larger values on these evaluation metrics than its counterpart in Tables 2–3 and Tables S3–S4. This observation suggests that shared GO annotations in one sub-ontology give clues of shared GO annotations in another sub-ontology. That is because the molecular function, biological roles and cellular location of gene products have some correlations. For this reason, the improvement between M+H →H and H →H is smaller than that in Tables 2–3. One exceptional observation is that *MacroAvgF1* is significantly reduced in CC and MF sub-ontology in Table 4. The reason is that BP sub-ontology have more general terms than that other sub-ontology. These general BP terms are annotated to much more genes than specific (or sparse) terms, so they often rank ahead of the terms in CC and MF sub-ontology, and are more likely being predicted as missing annotations of a gene by Eq. (7) or (Eq. (8)).

Overall, M+H →H significantly outperforms H →H and M →H, and H+M →M works much better than M →M and H →M, by Wilcoxon signed rank test with *p*<10^−10^. These superior results again corroborate the effectiveness of semantic similarity based interspecies gene function prediction by synergy GO annotations of genes from homologous species.

To further study that GO annotations of genes from two species with high homology are more complementary for each other than two species without such high homology, we conduct additional experiments on annotations of Yeast, Fly and Human using the similar protocol as in previous experiments. The results under different semantic similarity metrics are include in Tables S10(TO) and Table S11(BMA) of Additional file 1. From these tables, we can observe that F+Y →Y and Y+F →F always achieve better result than H+F →F and H+Y →Y, since Fly and Yeast share larger homology than that between Human. In summary, these comparative studies further confirm that it is more prominent to perform semantic similarity based interspecies gene function prediction across species with high homology than that with low homology.

### Prediction on simulated missing annotations

In this section, we perform simulated experiments by randomly masking a fixed number (*q*=1, 2, 3) annotations of a gene, and take these masked annotations as missing annotations of the gene. Next, we follow the similar procedure as in the previous experiments to replenish these missing annotations. From Fig. 1, we can see the terms annotated to a gene form a hierarchy by themselves. In the masking process, any leaf term in the hierarchy can be masked (or removed), once the descendant terms of a non-leaf term are all masked, then itself can also be masked. All these masked terms are viewed as simulated missing annotations of the gene. To avoid random effect of masked GO annotations, we repeat the experiments 10 times for each setting of *q*. The results (average of 10 independent repetitions and the standard deviation) are reported in Table 5 using semantic similarity BMA in CC sub-ontology and Table 6 in MF sub-ontology. Additional results with respect to other semantic similarities between genes are included in Tables S5–S9 of the Additional file 1. In these tables, the results in **bold** font are statistically better than their counterparts, according to pairwise *t*-test at 95% significance level.

**Table 5:** Prediction on simulated missing GO annotations under *BMA* in CC sub-ontology

*m*		MicroAvgF1	MacroAvgF1	1-RankLoss	Fmax	RAccuracy
1	H →H	96.03 ±0.09	86.84 ±0.19	96.49 ±0.02	95.36 ±0.09	17.12 ±1.89
	M+H →H	**96.77 ±0.01**	**86.86 ±0.23**	**97.20 ±0.01**	**95.55 ±0.01**	**32.55 ±0.16**
	M →M	95.48 ±0.04	86.19 ±0.22	93.83 ±0.01	94.98 ±0.04	12.22 ±0.73
	H+M →M	**96.57 ±0.06**	**86.33 ±0.26**	**97.23 ±0.26**	**95.15 ±0.06**	**33.28 ±1.11**
2	H →H	89.09 ±0.02	67.85 ±0.37	86.95 ±0.06	87.84 ±0.02	23.04 ±0.17
	M+H →H	**90.82 ±0.02**	**68.47 ±0.37**	**90.54 ±0.05**	**88.84 ±0.02**	**35.24 ±0.13**
	M →M	87.31 ±0.06	66.78 ±0.48	82.46 ±0.04	85.95 ±0.06	16.69 ±0.41
	H+M →M	**90.53 ±0.01**	**67.23 ±0.52**	**91.43 ±0.03**	**88.16 ±0.01**	**37.84 ±0.10**
3	H →H	82.54 ±0.06	53.68 ±0.25	79.52 ±0.02	81.76 ±0.06	25.08 ±0.27
	M+H →H	**85.87 ±0.05**	**54.78 ±0.27**	**86.17 ±0.09**	83.74 ±0.05	**39.38 ±0.20**
	M →M	81.45 ±0.05	52.84 ±0.55	76.54 ±0.07	77.69 ±0.05	24.71 ±0.19
	H+M →M	**84.02 ±0.10**	**53.83 ±0.56**	**82.41 ±0.12**	**79.60 ±0.10**	**35.13 ±0.41**

*q* is the number of simulated missing annotations of a gene. H →H directly uses GO annotations of Human to predict annotations of Human genes. M+H →H uses GO annotations of genes from Mouse and Human to predict annotations of Human genes. M →M and H+M →M follow the similar protocol, but make prediction for Mouse genes. The data in boldface is the statistically significant best among these comparing methods for a particular target species, and the significance is checked by paired t-test at 95% confidence intervals

**Table 6:** Prediction on simulated missing GO annotations under *BMA* in MF sub-ontology

*q*		MicroAvgF1	MacroAvgF1	1-RankLoss	Fmax	RAccuracy
1	H →H	91.71 ±0.02	82.31 ±0.22	90.98 ±0.13	91.37 ±0.02	10.56 ±0.21
	M+H →H	**93.70 ±0.02**	**82.33 ±0.21**	**96.91 ±0.03**	**93.87 ±0.02**	**32.30 ±0.26**
	M →M	92.01 ±0.10	80.50 ±0.41	93.04 ±0.02	92.71 ±0.10	7.85 ±1.15
	H+M →M	**93.09 ±0.02**	**80.60 ±0.40**	**96.78 ±0.03**	**93.27 ±0.02**	**19.59 ±0.21**
2	H →H	80.69 ±0.05	57.31 ±0.41	80.40 ±0.17	80.04 ±0.05	25.12 ±0.21
	M+H →H	**83.56 ±0.03**	**57.81 ±0.44**	**89.24 ±0.04**	**85.04 ±0.03**	**36.25 ±0.11**
	M →M	79.02 ±0.01	54.82 ±0.41	78.43 ±0.02	79.26 ±0.01	15.78 ±0.05
	H+M →M	**83.03 ±0.03**	**55.31 ±0.44**	**87.89 ±0.04**	**82.83 ±0.03**	**31.86 ±0.11**
3	H →H	70.09 ±0.03	40.45 ±0.42	68.16 ±0.03	70.32 ±0.03	23.70 ±0.08
	M +*H*→H	**74.76 ±0.04**	**41.37 ±0.39**	**80.44 ±0.04**	**76.58 ±0.04**	**35.62 ±0.11**
	M →M	68.89 ±0.08	39.27 ±0.25	65.40 ±0.10	68.25 ±0.08	20.59 ±0.21
	H+M →M	**73.68 ±0.04**	**40.18 ±0.33**	**76.63 ±0.07**	**72.99 ±0.04**	**32.81 ±0.09**

*q* is the number of simulated missing annotations of a gene. H →H directly uses GO annotations of Human to predict annotations of Human genes. M+H →H uses GO annotations of genes from Mouse and Human to predict annotations of Human genes. M →M and H+M →M follow the similar protocol, but make prediction for Mouse genes. The data in boldface is the statistically significant best among these comparing methods for a particular target species, and the significance is checked by paired t-test at 95% confidence intervals

From these tables, we can see M+H →H also achieves better results than H →H, and H+M →M outperforms M →M, irrespective of the sub-ontology, the setting value of *q* and the adopted semantic similarity. These results again support our motivation to synergy GO annotations and semantic similarity between genes from two homologous species, instead from single species. The improvement of M+H →H to H →H is more obvious than that on archived GO annotations as reported in the previous section. The cause is that the actual missing annotations of a gene often correspond to descents of several (or only one) terms annotated to the gene, instead of all the terms [26]. In contrast, our simulated experiment equally masks all leaf terms in the self-formed hierarchy of the gene. From the self-formed hierarchy of a gene and true path rule, we can see the masked terms of a gene are corresponding to specific terms, which are annotated to fewer genes than their ancestor terms. *MacroAvgF1* is biased toward specific terms, *MicroAvgF1* is biased toward non-specific terms, so the improvement of *MicroAvgF1* is more significant than that of *MacroAvgF1* in the simulated experiments.

In the end, we have to remark that GO annotations of gene products in recent GOA files are still not complete, all the reported results are conservative, since a predicted annotation not appear in the GOA file should not simply be taken as a false positive prediction. This predicted annotation may be lack of experimental evidences, or not curated by GO consortium, and thus it is not included into the GOA file by now. We also want to note that the studied semantic similarity based interspecies gene function prediction can only apply to genes with some annotations. Similar to other techniques, interspecies gene function prediction may result in over-annotated terms to genes. One possible way to mitigate this issue is to integrate with more biological data and work together with other techniques [53–55]. Synergy multiple types of biological data from different species, ontology hierarchy and semantic similarity to further boost the performance of interspecies gene function prediction is an interesting future pursue. We believe our work can prompt more work on semantic similarity based gene function prediction across species, especially for the species with high homology.

## Conclusions

In this paper, we investigate the possibility of predicting GO annotations of gene products across species using semantic similarity between genes. For this purpose, we adopt three widely used semantic similarity metrics and collect GO annotations of four species (Human, Mouse, Danio rerio and Arabidopsis thaliana). Our extensive experimental results show that interspecies gene function prediction using GO annotations of two highly homologous species is more prominent than that of two species without such high homology. Our investigation shows GO annotations of two homologous species are complementary for each other. However, for two species with low homology, it is not helpful to synergy their GO annotations for interspecies gene function prediction.

There are several avenues for future work. Adaptive setting *k*_1_ and *k*_2_ can further improve the accuracy of interspecies gene function prediction. Synergy the semantic similarity with other biological data can enhance functional association coherency between genes and thus to boost the prediction accuracy. Designing more advanced semantic similarity metric that takes into account incomplete and shallow annotations of genes is another interesting future pursue.

## Additional file

Additional file 1 Supplementary file of ‘Interspecies gene function prediction using semantic similarity’. This PDF file includes achieved GO annotations of hMAP4K2, hMAP4K2 and Map4k2 from Jan-2014 to Jan-2016, definition of evaluation metrics, and additional experimental results mentioned in the main text.
