# Supplementary material for: Interspecies gene function prediction using semantic similarity
Source: BMC Syst Biol. 2016 Dec 23;10(Suppl 4):121. doi: 10.1186/s12918-016-0361-5 (PMC5260010; doi:10.1186/s12918-016-0361-5)
Supplement: Additional file 1 — Supplementary file of ‘Interspecies gene function prediction using semantic similarity’. This PDF file includes achieved GO annotations of hMAP4K2, hMAP4K2 and Map4k2 from Jan-2014 to Jan-2016, definition of evaluation metrics, and additional experimental results mentioned in the main text. [file 12918_2016_361_MOESM1_ESM.pdf]

Supplementary file of 'Interspecies gene function prediction using semantic similarity'

Guoxian Yu*, Wei Luo, Guangyuan Fu, Jun Wang
College of Computer and Information Science, Southwest University, Chongqing 400715, China.
*Contact: gxyu@swu.edu.cn

August 6, 2016

1 GO annotations of hMAP4K2, hMAP4K2 and Map4k2

hMAP4K2, hMAP4K3 are two proteins from Human, and Map4k2 is a protein from Mouse. hMAP4K3 is a paralog of hMAP4K2, and Map4k2 is a ortholog of hMAP4K2. Table S1 records the evolution of GO annotations of these proteins from Jan-2014 to Jan-2016. From the table, we can see a number of missing annotations of Map4k2 were replenished by Jan-2016, and the terms corresponding to these missing annotations were already annotated to hMAP4K2 or hMAP4K3 by Jan-2014. We can also find a replenished missing annotation of hMAP4K2, and the corresponding term was already annotated to Map4k2 by Jan-2014.

Table S1: GO annotations of hMAP4K2, hMAP4K3 and Map4k2. 'x' means inferred annotation (inferred by true path rule); 'xx' means direct annotations; '**x**' and '**xx**' are missing annotation of Map4k2 by 2014 while hMAP4K2 or hMAP4K3 annotated with them; '**xx**' are the missing annotations of hMAP4K2 or hMAP4K3 by Jan-2014 while Map4k2 annotated with them.

GO term id	Jan-2014			Jan-2016		
	hMAP4K2	Map4k2	hMAP4K3	hMAP4K2	Map4k2	hMAP4K3
GO:0000165	x	x	x	x	x	x
GO:0000185	xx			xx		
GO:0000187	xx			x	x	
GO:0001932	x	x	x	x	x	x
GO:0001934	x	x	x	x	x	x
GO:0002376	x			x		
GO:0006464	x	x	x	x	x	x
GO:0006468	xx	xx	xx	xx	xx	xx
GO:0006793	x	x	x	x	x	x
GO:0006796	x	x	x	x	x	x
GO:0006810					x	
GO:0006903					xx	
GO:0006950	xx		x	x	x	x
GO:0006955	xx			xx		
GO:0007154	x	x	x	x	x	x
GO:0007165	x	x	x	x	x	x
GO:0007254	xx		xx	xx	x	xx
GO:0007257	xx			xx	xx	
GO:0007346		x		xx	xx	xx
GO:0008152	x	x	x	x	x	x
GO:0009314			x			x
GO:0009411			xx			xx
GO:0009416			x			x
GO:0009628			x			x
GO:0009893	x	x	x	x	x	x
GO:0009966	x	x	x	x	x	x
GO:0009967	x	x	x	x	x	x
GO:0009987	x	x	x	x	x	x
GO:0010033			x			x

GO term id	Jan-2014			Jan-2016		
	hMAP4K2	Map4k2	hMAP4K3	hMAP4K2	Map4k2	hMAP4K3
GO:0010562	x	x	x	x	x	x
GO:0010604	x	x	x	x	x	x
GO:0010646	x	x	x	x	x	x
GO:0010647	x	x	x	x	x	x
GO:0010941				x	x	x
GO:0016192					x	
GO:0016310	x	x	x	x	x	x
GO:0019220	x	x	x	x	x	x
GO:0019222	x	x	x	x	x	x
GO:0019538	x	x	x	x	x	x
GO:0023014	x	x	x	x	xx	x
GO:0023051	x	x	x	x	x	x
GO:0023052	x	x	x	x	x	x
GO:0023056	x	x	x	x	x	x
GO:0031098	x		x	x	xx	x
GO:0031323	x	x	x	x	x	x
GO:0031325	x	x	x	x	x	x
GO:0031399	x	x	x	x	x	x
GO:0031401	x	x	x	x	x	x
GO:0032147	x	x	x	x	xx	x
GO:0032268	x	x	x	x	x	x
GO:0032270	x	x	x	x	x	x
GO:0032872	x			x	**x**	
GO:0032874	x	x		x	x	
GO:0033554	x		x	x	**x**	x
GO:0033674	x		x	x	**x**	x
GO:0034097			x			x
GO:0034612			xx			x
GO:0035556	xx	xx	xx	xx	xx	x
GO:0036211	x	x	x	x	x	x
GO:0042221			x			x
GO:0042325	x	x	x	x	x	x
GO:0042327	x	x	x	x	x	x
GO:0042981				xx	xx	xx
GO:0043067				x	x	x
GO:0043085	x	x	x	x	x	x
GO:0043170	x	x	x	x	x	x
GO:0043405	x	x	x	x	x	x
GO:0043406	x			x	**x**	
GO:0043408	x	x	x	x	x	x
GO:0043410	x	x	x	x	x	x
GO:0043412	x	x	x	x	x	x
GO:0043506	x			x	**x**	
GO:0043507	x			x	**x**	
GO:0043549	x	x	x	x	x	x
GO:0044093	x	x	x	x	x	x
GO:0044237	x	x	x	x	x	x
GO:0044238	x	x	x	x	x	x
GO:0044260	x	x	x	x	x	x
GO:0044267	x	x	x	x	x	x
GO:0044699	x	x	x	x	x	x
GO:0044700	x	x	x	x	x	x
GO:0044710	x	x	x	x	x	x
GO:0044763	x	x	x	x	x	x
GO:0045859	x	x	x	x	x	x
GO:0045860	x	x	x	x	x	x
GO:0045937	x	x	x	x	x	x
GO:0046328	x			x	**x**	
GO:0046330	xx			xx	**xx**	

GO term id	Jan-2014			Jan-2016		
	hMAP4K2	Map4k2	hMAP4K3	hMAP4K2	Map4k2	hMAP4K3
GO:0048518	x	x	x	x	x	x
GO:0048522	x	x	x	x	x	x
GO:0048583	x	x	x	x	x	x
GO:0048584	x	x	x	x	x	x
GO:0050789	x	x	x	x	x	x
GO:0050790	x	x	x	x	x	x
GO:0050794	x	x	x	x	x	x
GO:0050896	x	x	x	x	x	x
GO:0051174	x	x	x	x	x	x
GO:0051179					x	
GO:0051234					x	
GO:0051640					x	
GO:0051641					x	
GO:0051648					x	
GO:0051649					x	
GO:0051650					x	
GO:0051656					x	
GO:0051246	x	x	x	x	x	x
GO:0051247	x	x	x	x	x	x
GO:0051338	x	x	x	x	x	x
GO:0051347	x	x	x	x	x	x
GO:0051403	x		x	x	x	x
GO:0051716	x	x	x	x	x	x
GO:0051726				x	x	x
GO:0060255	x	x	x	x	x	x
GO:0065007	x	x	x	x	x	x
GO:0065009	x	x	x	x	x	x
GO:0070302	x			x	x	
GO:0070304	x			x	x	x
GO:0071704	x	x	x	x	x	
GO:0071900	x	x	x	x	x	x
GO:0071902	x	x	x	x	x	x
GO:0080090	x	x	x	x	x	x
GO:0080134	x			x	x	
GO:0080135	x			x	x	
GO:1902531	x	x	x	x	x	x
GO:1902533	x	x	x	x	x	x

Table S2: The sources and evidence codes of new overlapped annotations of hMAP4K2, Map4k2, hMAP4K3 from Jan-2014 to Jan-2016.

Protein	GO terms	Sources	Evidence
hMAP4K2	GO:0007346	GO:0000033 PANTHER:PTN000684825	IBA
hMAP4K3	GO:0007346	GO:0000033 PANTHER:PTN000684825	IBA
Map4k2	GO:0000187	PMID:15896720	IMP
	GO:0006950	PMID:9135144	IDA
	GO:0032872	PMID:19565474	TAS
	GO:0033674	GO:0000096 UniProtKB:O60934	ISO
	GO:0043507	PMID:10523642	IDA
	GO:0046328	GO:0000096 UniProtKB:P54762	ISO
	GO:0007257	GO:0000096 UniProtKB:Q12851	ISO
	GO:00046330	GO:0000096 UniProtKB:Q12851	ISO
	GO:00051403	GO:0000096 UniProtKB:Q9BWF3	ISO
	GO:00070304	GO:0000096 UniProtKB:P07948	ISO

2 Evaluation Metrics

In the main text, we introduce five evaluation metrics, which are used to compare performance of multi-label learning [3] and function prediction across different methods [1, 2]. The formal definition of these metrics are described as below.

MacroAvgF1 is a term-centric metrics, it averages $F1$ scores of T different terms:

$$MacroAvgF1 = \frac{1}{T}\sum_{t=1}^{T}\frac{2p_t r_t}{p_t + r_t}$$

where p_t and r_t are the precision and recall of term t. *MacroAvgF1* first calculates $F1$ scores for each term and then averages over all the terms. It is biased toward specific terms that annotated to few gene products.

MicroAvgF1 is another term-centric metric, it computes the $F1$ measure on the predictions of different terms as a whole:

$$MicroAvgF1 = \frac{\sum_{t=1}^{T} 2p_t r_t}{\sum_{t=1}^{T} p_t + r_t}$$

MicroAvgF1 is more bias toward the general terms that annotated to a number of gene products.

RankLoss is a protein-centric metric, it computes the average fraction of not correctly ranked predicted term pairs as follow:

$$RankLoss = \frac{1}{N}\sum_{i=1}^{N}\frac{1}{|\mathcal{T}_i||\bar{\mathcal{T}}_i|}|\mathcal{R}_i|$$

where $\mathcal{R}_i = \{(t_1, t_2) \in \mathcal{T}_i \times \bar{\mathcal{T}}_i | p(i, t_1) \leq p(i, t_2)\}$, $\bar{\mathcal{T}}_i$ is the complement set of $\mathcal{T}_i$. The performance is perfect when *RankLoss=0*. In this case, the predicted likelihood of any $t_1 \in \mathcal{T}_i$ is larger than that $t_2 \in \bar{\mathcal{T}}_i$. The smaller the value of *RankLoss*, the better the performance.

Fmax is a protein-centric evaluation metric introduced in Critical Assessment of protein Function Annotation (CAFA) [1], *Fmax* is an F-measure computed as:

$$Fmax = \max_{\tau}\frac{2 \cdot pr(\tau) \cdot rc(\tau)}{pr(\tau) + rc(\tau)}$$

where $pr(\tau) = \frac{1}{m(\tau)}\sum_{i=1}^{N(\tau)} pr_i(\tau)$ is the the precision at threshold $\tau \in [0, 1]$, $pr_i(\tau)$ is the precision on the i-th gene, $N(\tau)$ is the number of genes on which at least one prediction was made above the threshold τ, $rc(\tau) = \frac{1}{N}\sum_{i=1}^{N} rc_i(\tau)$ is the recall across N genes at threshold τ. To find the maximum *Fmax*, we vary τ from 0 to 1 with stepsize 0.02.

RAccuracy [2] evaluates how many missing annotations of N genes are correctly predicted. Suppose the predicted function annotation set of the i-th gene is $\mathcal{T}_i^p$, the annotation set from historical GOA file for the gene is $\mathcal{T}_i^h$, and updated annotation set from recent GOA file is $\mathcal{T}_i^r$. *RAccuracy* is computed as follows:

$$RAccuracy = \frac{\sum_{i=1}^{N}|(\mathcal{T}_i^r - \mathcal{T}_i^h) \cap \mathcal{T}_i^p|}{\sum_{i=1}^{N}|T_i^r - T_i^h|}$$

where $|\mathcal{T}_i^r - \mathcal{T}_i^h|$ counts missing annotations of the i-th gene, and $|(\mathcal{T}_i^r - \mathcal{T}_i^h) \cap \mathcal{T}_i^p|$ counts correctly predicted missing annotations.

MacroAvgF1, *MicroAvgF1* and *RAccuracy* require to transform the predicted likelihoods $p(i, \cdot) \in \mathbb{R}^T$ in Eq. (7) (or Eq. (8) in the main text, into a binary indicative vector, here we choose the terms corresponding to the largest m entries of $p(i, \cdot)$ as the predicted annotations of the i-th gene, and m is equal to $|\mathcal{T}_i^r|$.

3 Results on Archived GOA files

Tables S11-S4 include the results on archived GOA files using semantic similarity *simGIC*, and *simGICs* (see Eq. (6) in the main text) with information content of a term is computed by Eq. (3) in the main text. Since simGIC and simGIC take longer to finish than other comparing metrics, we do not include the results of M→H, D→H, and A→H. However, our preliminary results also give the similar patterns as that using BAM and TO. **Table S5 report the results** on archived GOA files using semantic similarity *TO* (see Eq. (5) in main text) by combining the GO annotations in CC, MF and BP together and then evaluating in each sub-ontology. Obviously, these results provide the similar observations and conclusions as in the main text.

Table S3: Prediction on archived GOA files using *simGIC* (see Eq. (6) in the main text). H→H directly uses GO annotations of Human to predict annotations of Human genes. M+H→H uses GO annotations of genes from Mouse and Human to predict annotations of Human genes. D+H→H uses GO annotations of genes from Danio rerio and Human to predict annotations of Human genes. A+H→H uses annotations of genes from Arabidopsis thaliana and Human to predict annotations of Human genes. M→M, H+M→M, D+M→M and A+M→M follow the similar protocol, but make prediction for Mouse genes.

		MicroAvgF1	MacroAvgF1	1-RankLoss	Fmax	RAccuracy
CC	H→H	0.8435	0.7301	0.9048	0.8775	0.2080
	M→H	0.8408	0.7188	0.9045	0.8745	0.1946
	M+H→H	**0.8596**	**0.7633**	**0.9758**	**0.8764**	**0.2898**
	D→H	0.8445	0.7219	0.9077	0.8571	0.1940
	D+H→H	0.8554	0.7438	0.9481	0.8619	0.2686
	A→H	0.8381	0.7320	0.8883	0.8545	0.1811
	A+H→H	0.8427	0.7304	0.9055	0.8790	0.2042
	M→M	0.7898	0.6198	0.8981	0.8565	0.2591
	H→M	0.7818	0.6114	0.8939	0.8388	0.2562
	H+M→M	**0.8167**	**0.6561**	**0.9613**	**0.8657**	**0.3541**
	D→M	0.7892	0.6141	0.8961	0.8373	0.2277
	D+M→M	0.8070	0.6312	0.9317	0.8386	0.3197
	A→M	0.7804	0.6264	0.8686	0.8356	0.2264
	A+M→M	0.7893	0.6190	0.8955	0.8426	0.2574
MF	H→H	0.8632	0.8239	0.9383	0.8885	0.2051
	M→H	0.8620	0.8184	0.9359	0.8626	0.2001
	M+H→H	**0.8738**	**0.8380**	**0.9769**	**0.9040**	**0.2665**
	D→H	0.8590	0.8166	0.9288	0.8846	0.1807
	D+H→H	0.8694	0.8352	0.9630	0.8977	0.2411
	A→H	0.8486	0.8140	0.9098	0.8936	0.1201
	A+H→H	0.8591	0.8172	0.9332	0.8930	0.1811
	M→M	0.7842	0.6929	0.9061	0.8655	0.2015
	H→M	0.7864	0.6880	0.9044	0.8481	0.2071
	H+M→M	0.7885	**0.7105**	0.9387	**0.8753**	0.2175
	D→M	0.7833	0.7031	0.9310	0.8444	0.1984
	D+M→M	**0.7904**	0.7082	**0.9407**	0.8701	**0.2244**
	A→M	0.7671	0.6797	0.8735	0.8603	0.1383
	A+M→M	0.7810	0.6803	0.9047	0.8610	0.1897
BP	H→H	0.8499	0.8031	0.9698	0.7899	0.2660
	M→H	0.8458	0.7972	0.9600	0.7913	0.2461
	M+H→H	**0.8596**	**0.7633**	**0.9758**	**0.8337**	**0.2898**
	D→H	0.8396	0.8012	0.9559	0.7917	0.2157
	D+H→H	0.8469	0.8023	0.9685	0.8016	0.2514
	A→H	0.8312	0.7892	0.9298	0.7651	0.1747
	A+H→H	0.8424	0.7954	0.9620	0.7873	0.2293
	M→M	0.8019	0.7122	0.9636	0.7852	0.2624
	H→M	0.8032	0.7033	0.9571	0.7811	0.2673
	H+M→M	**0.8052**	**0.7162**	**0.9703**	**0.8229**	**0.2749**
	D→M	0.7903	0.7112	0.9458	0.7756	0.2193
	D+M→M	0.7978	0.7121	0.9611	0.7916	0.2472
	A→M	0.7861	0.6922	0.9326	0.7542	0.1962
	A+M→M	0.7934	0.7045	0.9554	0.7676	0.2308

4 Results on Simulated Missing Annotations

Tables S6-S9 include additional experimental results on simulated missing annotations. Since the simulated experiments repeat for 10 times for each fixed setting and ask for long time, especially for BP sub-ontology, which includes much more terms., we do not report the results with respect to the comparing methods as in the previous section. The preliminary results are consistent with the results reported in **Table S6-S9.** These results also give the similar observations and conclusion as discussed in the main text.

Table S4: Prediction on archived GOA files using *simGICs* (see Eq. (6) in the main text) with information content of a term is computed by Eq. (3). H→H directly uses GO annotations of Human to predict annotations of Human genes. M→H only employs annotations of genes from Mouse to predict annotations of Human genes. M+H→H uses GO annotations of genes from Mouse and Human to predict annotations of Human genes. D+H→H uses annotations of genes from Danio rerio and Human to predict annotations of Human genes. A+H→H uses annotations of genes from Arabidopsis thaliana and Human to predict annotations of Human genes. M→M, H+M→M, D+M→M and A+M→M follow the similar protocol, but predict annotations of Mouse genes.

		MicroAvgF1	MacroAvgF1	1-RankLoss	Fmax	RAccuracy
CC	H→H	0.8555	0.7363	0.9239	0.8773	0.2688
	M+H→H	**0.8601**	**0.7638**	**0.9746**	**0.8791**	**0.2923**
	D+H→H	0.8578	0.7562	0.9493	0.8757	0.2765
	A+H→H	0.8559	0.7397	0.9277	0.8775	0.2710
	M→M	0.8023	0.6330	0.9178	0.8568	0.3064
	H+M→M	**0.8195**	**0.6581**	**0.9695**	**0.8650**	**0.3639**
	D+M→M	0.8046	0.6304	0.9294	0.8562	0.3199
	A+M→M	0.8040	0.6327	0.9146	0.8438	0.3012
MF	H→H	0.8652	0.8244	0.8652	0.8894	0.2631
	M+H→H	**0.8747**	**0.8385**	**0.8747**	**0.9051**	**0.3152**
	D+H→H	0.8501	0.8251	0.8570	0.8751	0.2455
	A+H→H	0.8314	0.8103	0.8415	0.8645	0.2061
	M→M	0.7885	0.6908	0.7885	0.8688	0.2177
	H+M→M	**0.7890**	**0.7091**	**0.7890**	**0.8756**	**0.2193**
	D+M→M	0.7885	0.7037	0.7869	0.8679	0.2148
	A+M→M	0.7858	0.6828	0.7870	0.8682	0.2075
BP	H→H	0.8511	0.8039	0.9740	0.7911	0.2722
	M+H→H	**0.8518**	**0.8066**	**0.9772**	**0.8342**	**0.2755**
	D+H→H	0.8482	0.8033	0.9732	0.8010	0.2576
	A+H→H	0.8440	0.7965	0.9674	0.7905	0.2372
	M→M	0.8048	0.7138	0.9708	0.7875	0.2732
	H+M→M	**0.8064**	**0.7164**	**0.9717**	**0.8227**	**0.2792**
	D+M→M	0.8006	0.7135	0.9680	0.7904	0.2576
	A+M→M	0.7957	0.7058	0.9629	0.7721	0.2393

References

[1] Radivojac, P and Clark, W T and Oron, T R *et al.* *A large-scale evaluation of computational protein function prediction*, Nature Methods, 2013, 10(3), 221-227.

[2] Yu, G and Domeniconi, C and Rangwala, H and Zhang, G. *Protein function prediction using dependence maximization*, in Proceedings of European Conference on Machine Learning and Knowledge Discovery in Databases(ECML/PKDD), 2013, 574–589.

[3] Zhang, M and Zhou, Z. *A review on multi-label learning algorithms*, IEEE Transactions on Knowledge and Data Engineering, 2014, 26(8), 1819–1837.

Table S5: Prediction on archived GOA files using *TO* (see Eq. (6)) by combining the GO annotations in CC, MF and BP together and then evaluating in each sub-ontology. H→H directly uses GO annotations of Human to predict annotations of Human genes. M→H only employs annotations of genes from Mouse to predict annotations of Human genes. M+H→H uses GO annotations of genes from Mouse and Human to predict annotations of Human genes. D+H→H uses annotations of genes from Danio rerio and Human to predict annotations of Human genes. A+H→H uses annotations of genes from Arabidopsis thaliana and Human to predict annotations of Human genes. M→M, H+M→M, D+M→M and A+M→M follow the similar protocol, but predict annotations of Mouse genes.

		MicroAvgF1	MacroAvgF1	1-RankLoss	Fmax	RAccuracy
CC	H→H	0.8798	0.4449	0.9777	0.8435	0.2558
	M→H	0.8735	0.4423	0.9732	0.8574	0.2275
	M+H→H	**0.8806**	**0.4459**	**0.9834**	**0.8948**	**0.2908**
	D→H	0.8657	0.4443	0.9722	0.8904	0.1795
	D+H→H	0.8764	0.4444	0.9788	0.8658	0.2449
	A→H	0.8585	0.4380	0.9487	0.8559	0.1355
	A+H→H	0.8715	0.4406	0.9721	0.8537	0.2152
	M→M	0.8312	0.4505	0.9728	0.8447	0.2115
	H→M	0.8301	0.4478	0.9713	0.8307	0.2100
	H+M→M	**0.8330**	**0.4523**	**0.9785**	**0.8805**	**0.2195**
	D→M	0.8187	0.4517	0.9649	0.8677	0.1528
	D+M→M	0.8255	0.4504	0.9729	0.8521	0.1848
	A→M	0.8119	0.4438	0.9262	0.8234	0.1212
	A+M→M	0.8259	0.4459	0.9656	0.8369	0.1865
MF	H→H	0.8619	0.4305	0.9706	0.8182	0.2421
	M→H	0.8563	0.4290	0.9665	0.8004	0.2114
	M+H→H	**0.8633**	**0.4318**	**0.9798**	**0.8787**	**0.2500**
	D→H	0.8486	0.4310	0.9627	0.8386	0.1690
	D+H→H	0.8582	0.4300	0.9700	0.8350	0.2219
	A→H	0.8397	0.4265	0.9311	0.7671	0.1202
	A+H→H	0.8504	0.4269	0.9629	0.8029	0.1788
	M→M	0.8097	0.4075	0.9665	0.8103	0.2015
	H→M	0.8114	0.4043	0.9637	0.8100	0.2086
	H+M→M	**0.8150**	**0.4104**	**0.9759**	**0.8650**	**0.2239**
	D→M	0.7996	0.4094	0.9550	0.8280	0.1591
	D+M→M	0.8059	0.4084	0.9637	0.8235	0.1856
	A→M	0.7885	0.4044	0.9196	0.7331	0.1126
	A+M→M	0.8000	0.4040	0.9564	0.7676	0.1609
BP	H→H	0.8467	0.8021	0.9623	0.7868	0.2505
	M→H	0.8432	0.7985	0.9566	0.7834	0.2333
	M+H→H	**0.8503**	**0.8071**	**0.9748**	**0.8332**	**0.2679**
	D→H	0.8385	0.8025	0.9577	0.7884	0.2101
	D+H→H	0.8450	0.8018	0.9631	0.7975	0.2422
	A→H	0.8305	0.7921	0.9304	0.7585	0.1711
	A+H→H	0.8395	0.7935	0.9540	0.7815	0.2153
	M→M	0.7970	0.7108	0.9542	0.7805	0.2444
	H→M	0.7956	0.7049	0.9504	0.7773	0.2401
	H+M→M	**0.8033**	**0.7163**	**0.9682**	**0.8226**	**0.2675**
	D→M	0.7888	0.7126	0.9473	0.7751	0.2138
	D+M→M	0.7946	0.7110	0.9532	0.7835	0.2352
	A→M	0.7789	0.6999	0.9122	0.7406	0.1769
	A+M→M	0.7892	0.7020	0.9439	0.7609	0.2152

Table S6: Prediction on simulated missing GO annotations under *TO* (see Eq. (6)) in CC sub-ontology. q is the number of simulated missing annotations of a gene. H→H directly uses GO annotations of genes from Human to predict missing annotations. M→H uses annotations of genes from Mouse and Human to predict missing annotations of Human genes.

q		MicroAvgF1	MacroAvgF1	1-RankLoss	Fmax	RAccuracy
1	H→H	91.98±0.07	82.48±0.21	92.54±0.03	91.48±0.07	13.54±0.77
	M+H→H	**94.33±0.02**	**82.53±0.21**	**99.19±0.01**	**94.32±0.02**	**38.88±0.26**
	M→M	92.53±0.02	80.69±0.41	95.57±0.08	92.86±0.02	13.05±0.20
	H+M→M	**93.98±0.02**	**80.74±0.41**	**98.94±0.01**	**93.35±0.02**	**29.99±0.25**
2	H→H	79.51±0.06	57.65±0.45	76.31±0.10	75.75±0.06	20.53±0.21
	M+H→H	**83.47±0.02**	**57.93±0.43**	**94.86±0.04**	**80.38±0.02**	**35.90±0.10**
	M→M	79.50±0.04	55.11±0.45	79.63±0.11	77.88±0.04	17.70±0.17
	H+M→M	**83.98±0.07**	**55.45±0.45**	**93.52±0.02**	**82.79±0.07**	**35.69±0.28**
3	H→H	67.84±0.02	40.81±0.42	62.22±0.03	64.51±0.02	17.95±0.05
	M+H→H	**73.66±0.03**	**41.35±0.42**	**85.83±0.05**	**72.20±0.03**	**32.80±0.08**
	M→M	67.12±0.09	39.42±0.28	62.59±0.07	64.60±0.09	16.07±0.23
	H+M→M	**73.76±0.02**	**40.03±0.30**	**82.02±0.01**	**70.84±0.02**	**33.03±0.06**

Table S7: Prediction on simulated missing GO annotations under *TO* (see Eq. (6)) in MF sub-ontology. q is the number of simulated missing annotations of a gene. H→H directly uses GO annotations of genes from Human to predict missing annotations. M→H uses annotations of genes from Mouse and Human to predict missing annotations of Human genes.

q		MicroAvgF1	MacroAvgF1	1-RankLoss	Fmax	RAccuracy
1	H→H	96.63±0.12	87.04±0.23	99.38±0.01	95.98±0.12	29.74±2.60
	M+H→H	**97.53±0.02**	**87.18±0.23**	**99.90±0.00**	**96.58±0.02**	**48.51±0.50**
	M→M	96.01±0.06	86.36±0.21	97.84±0.25	95.50±0.06	22.43±1.12
	H+M→M	**97.28±0.01**	**86.63±0.08**	**99.87±0.00**	**95.60±0.01**	**46.99±0.24**
2	H→H	90.05±0.02	68.09±0.35	91.20±0.04	88.59±0.02	29.86±0.17
	M+H→H	**92.51±0.03**	**68.35±0.34**	**96.71±0.01**	**90.14±0.03**	**47.21±0.19**
	M→M	88.69±0.06	67.11±0.43	88.08±0.06	86.52±0.06	25.75±0.40
	H+M→M	**90.82±0.01**	**67.50±0.38**	**93.46±0.01**	**87.94±0.01**	**39.72±0.07**
3	H→H	84.74±0.03	54.08±0.25	84.33±0.11	81.41±0.03	34.53±0.14
	M+H→H	**87.36±0.05**	**54.47±0.21**	**89.33±0.11**	**83.38±0.05**	**45.75±0.23**
	M→M	83.02±0.03	52.83±0.34	80.31±0.02	79.11±0.03	31.08±0.11
	H+M→M	**85.56±0.02**	**53.20±0.78**	**85.32±0.04**	**81.04±0.02**	**41.40±0.10**

Table S8: Prediction on simulated missing GO annotations under *simGIC* in CC sub-ontology. q is the number of simulated missing annotations of a gene. H→H directly uses GO annotations of genes from Human to predict missing annotations. M→H uses annotations of genes from Mouse and Human to predict missing annotations of Human genes.

q		MicroAvgF1	MacroAvgF1	1-RankLoss	Fmax	RAccuracy
1	H→H	96.43±0.12	86.97±0.15	99.12±0.02	95.98±0.12	25.42±2.55
	M+H→H	**97.46±0.01**	**87.06±0.16**	**98.93±0.01**	**96.44±0.01**	**46.89±0.29**
	M→M	96.11±0.06	86.64±0.38	97.42±0.10	95.51±0.06	24.33±1.08
	H+M→M	**97.37±0.03**	**86.83±0.38**	**99.63±0.02**	**95.95±0.03**	**48.81±0.57**
2	H→H	90.31±0.02	68.02±0.47	91.60±0.06	88.22±0.02	31.66±0.16
	M+H→H	**92.77±0.04**	**68.48±0.47**	**95.77±0.03**	**90.43±0.04**	**48.99±0.29**
	M→M	88.87±0.05	67.23±0.41	87.25±0.10	86.50±0.05	26.93±0.34
	H+M→M	**91.31±0.05**	**67.66±0.40**	**92.13±0.04**	**89.02±0.05**	**42.98±0.32**
3	H→H	84.42±0.06	54.36±0.50	84.40±0.05	81.29±0.06	33.16±0.24
	M+H→H	**88.05±0.03**	**55.18±0.51**	**89.70±0.09**	**83.78±0.03**	**48.72±0.11**
	M→M	82.77±0.08	53.36±0.30	80.13±0.16	79.13±0.08	30.07±0.32
	H+M→M	**86.19±0.04**	**54.12±0.31**	**86.42±0.04**	**81.56±0.04**	**43.94±0.15**

Table S9: Prediction on simulated missing GO annotations under *simGIC* in MF sub-ontology. q is the number of simulated missing annotations of a gene. H→H directly uses GO annotations of genes from Human to predict missing annotations. M→H uses annotations of genes from Mouse and Human to predict missing annotations of Human genes.

q		MicroAvgF1	MacroAvgF1	1-RankLoss	Fmax	RAccuracy
1	H→H	92.07±0.07	82.48±0.21	92.35±0.01	91.48±0.07	14.48±0.77
	M+H→H	**94.48±0.03**	**82.53±0.22**	**98.51±0.01**	**94.11±0.03**	**40.46±0.33**
	M→M	92.67±0.11	80.68±0.39	95.09±0.13	92.86±0.11	14.70±1.23
	H+M→M	**94.15±0.03**	**80.79±0.42**	**98.62±0.00**	**93.06±0.03**	**31.93±0.37**
2	H→H	79.68±0.08	57.70±0.43	76.42±0.04	75.78±0.08	21.22±0.30
	M+H→H	**85.79±0.04**	**58.26±0.44**	**92.60±0.04**	**85.38±0.04**	**44.92±0.15**
	M→M	79.95±0.03	55.20±0.44	79.64±0.04	77.83±0.03	19.50±0.11
	H+M→M	**84.53±0.04**	**55.73±0.41**	**90.69±0.03**	**83.03±0.04**	**37.87±0.17**
3	H→H	68.63±0.06	40.93±0.43	63.11±0.03	65.61±0.06	19.97±0.15
	M+H→H	**77.55±0.01**	**41.90±0.42**	**83.51±0.03**	**77.51±0.01**	**42.73±0.02**
	M→M	67.68±0.07	39.51±0.28	63.34±0.07	65.01±0.07	17.51±0.17
	H+M→M	**75.90±0.03**	**40.58±0.31**	**80.01±0.04**	**73.59±0.03**	**38.48±0.08**

Table S10: Prediction on archived GOA files using *simTO* (see Eq. (6) in the main text). Y→Y directly uses GO annotations of Yeast to predict annotations of Yeast genes. F+Y→Y uses GO annotations of genes from Fly and Yeast to predict annotations of Yeast genes. H+Y→Y uses GO annotations of genes from Human and Yeast to predict annotations of Yeast genes. F→F, Y+F→F and H+F→F follow the similar protocol, but make prediction for Fly genes.

		MicroAvgF1	MacroAvgF1	1-RankLoss	Fmax	RAccuracy
CC	Y→Y	0.9578	0.8787	0.9854	0.9193	0.2817
	F→Y	0.9572	0.8750	0.9813	0.9248	0.2715
	F+Y→Y	**0.9616**	**0.8844**	**0.9927**	**0.9427**	**0.3471**
	H→Y	0.9513	0.8638	0.9686	0.9304	0.1862
	H+Y→Y	0.9551	0.8666	0.9791	0.9434	0.2365
	F→F	0.9182	0.7443	0.9733	0.8940	0.3275
	Y→F	0.9107	0.7393	0.9707	0.8467	0.3077
	Y+F→F	**0.9194**	**0.7559**	**0.9858**	**0.9150**	**0.3470**
	H→F	0.9148	0.7295	0.9643	0.9070	0.2991
	H+F→F	0.9149	0.7321	0.9627	0.8835	0.3042
MF	Y→Y	0.9220	0.8937	0.9745	0.9224	0.1776
	F→Y	0.9216	0.8794	0.9743	0.9161	0.1734
	F+Y→Y	**0.9258**	**0.8991**	**0.9856**	**0.9316**	**0.2180**
	H→Y	0.9156	0.8850	0.9715	0.8978	0.1096
	H+Y→Y	0.9199	0.8787	0.9741	0.9053	0.1552
	F→F	0.8947	0.7788	0.9672	0.8990	0.1840
	Y→F	0.8940	0.7756	0.9630	0.9076	0.1783
	Y+F→F	**0.8974**	**0.7813**	**0.9780**	**0.9315**	**0.2047**
	H→F	0.8879	0.7672	0.9665	0.9023	0.1308
	H+F→F	0.8941	0.7701	0.9665	0.9048	0.1790
BP	Y→Y	0.9112	0.8660	0.9843	0.8564	0.1810
	F→Y	0.9077	0.8648	0.9781	0.8335	0.1489
	F+Y→Y	**0.9140**	**0.8674**	**0.9870**	**0.9020**	**0.2065**
	H→Y	0.9120	0.8607	0.9783	0.8581	0.1881
	H+Y→Y	0.9138	0.8610	0.9819	0.8693	0.2048
	F→F	0.8606	0.7788	0.9622	0.7580	0.1765
	Y→F	0.8598	0.7755	0.9511	0.7551	0.1714
	Y+F→F	**0.8654**	**0.7822**	**0.9716**	**0.8201**	**0.2049**
	H→F	0.8619	0.7732	0.9595	0.7628	0.1837
	H+F→F	0.8613	0.7726	0.9574	0.7749	0.1801

Table S11: Prediction on archived GOA files using *simBMA* (see Eq. (6) in the main text). Y→Y directly uses GO annotations of Yeast to predict annotations of Yeast genes. F+Y→Y uses GO annotations of genes from Fly and Yeast to predict annotations of Yeast genes. H+Y→Y uses GO annotations of genes from Human and Yeast to predict annotations of Yeast genes. F→F, Y+F→F and H+F→F follow the similar protocol, but make prediction for Fly genes.

		MicroAvgF1	MacroAvgF1	1-RankLoss	Fmax	RAccuracy
CC	Y→Y	0.9606	0.8766	0.9857	0.9196	0.3305
	F→Y	0.9572	0.8667	0.9727	0.9300	0.2713
	F+Y→Y	**0.9617**	**0.8850**	**0.9921**	**0.9320**	**0.3492**
	H→Y	0.9514	0.8654	0.9628	0.9438	0.1931
	H+Y→Y	0.9576	0.8646	0.9749	0.9455	0.2787
	F→F	0.9155	0.7364	0.9552	0.8888	0.3055
	Y→F	0.9199	0.7358	0.9543	0.8427	0.3013
	Y+F→F	**0.9204**	**0.7579**	**0.9828**	**0.9127**	**0.3454**
	H→F	0.9127	0.7334	0.9502	0.9066	0.2830
	H+F→F	0.9138	0.7358	0.9512	0.8860	0.2961
MF	Y→Y	0.9238	0.8935	0.9734	0.9128	0.1968
	F→Y	0.9198	0.8778	0.9723	0.9008	0.1547
	F+Y→Y	**0.9257**	**0.8981**	**0.9834**	**0.9305**	**0.2166**
	H→Y	0.9177	0.8829	0.9688	0.9015	0.1318
	H+Y→Y	0.9206	0.8759	0.9717	0.8983	0.1624
	F→F	0.8940	0.7670	0.9612	0.8982	0.1781
	Y→F	0.8965	0.7699	0.9559	0.9026	0.1949
	Y+F→F	**0.8961**	**0.7822**	**0.9752**	**0.9275**	**0.1978**
	H→F	0.8919	0.7805	0.9645	0.8821	0.1617
	H+F→F	0.8936	0.7642	0.9580	0.8929	0.1756
BP	Y→Y	0.9093	0.8599	0.9757	0.8699	0.1635
	F→Y	0.9077	0.8648	0.9818	0.8552	0.1490
	F+Y→Y	**0.9115**	**0.8666**	**0.9848**	**0.9000**	**0.1835**
	H→Y	0.9046	0.8627	0.9738	0.8649	0.1199
	H+Y→Y	0.9090	0.8601	0.9775	0.8745	0.1610
	F→F	0.8570	0.7737	0.9479	0.7624	0.1551
	Y→F	0.8562	0.7712	0.9533	0.7829	0.1502
	Y+F→F	**0.8619**	**0.7811**	**0.9686**	**0.8335**	**0.1837**
	H→F	0.8520	0.7754	0.9491	0.7850	0.1255
	H+F→F	0.8542	0.7681	0.9460	0.7872	0.1384
